# Sarcome de kaposi et myélome multiple: s´agit-il d´une association causée par le HHV-8?

**DOI:** 10.11604/pamj.2020.36.85.22407

**Published:** 2020-06-12

**Authors:** Sophia Kahouli, Hafid Zahid, Majid Benkirane, Nezha Messaoudi

**Affiliations:** 1 Laboratoire de Recherche et d´Analyses Médicales de la Fraternelle de la Gendarmerie Royale, Rabat, Maroc,; 2 Faculté de Médecine et de Pharmacie de Rabat, Rabat, Maroc,; 3 Service d´Hématologie, Immunohématologie de l´Hôpital Militaire d´Instruction Mohammed V, Rabat, Maroc,; 4 Pôle des Laboratoires de l´Hôpital Militaire d´Instruction Mohammed V, Rabat, Maroc

**Keywords:** Sarcome de kaposi, myélome multiple, HHV-8, Kaposi´s sarcoma, multiple myeloma, HHV-8

## Abstract

Le sarcome de kaposi est une affection tumorale, caractérisé par des lésions violacées ou brunâtres sur la peau. Il s´agit d´un cancer le plus souvent lié à l´infection par l´herpès virus humain type 8 et peut être secondaire à une hémopathie maligne notamment les lymphomes. Nous rapportons à travers notre observation un autre nouveau cas qui illustre l´association très rare kaposi-myélome multiple. Mr âgé de 67 ans, suivi en dermatologie pour la maladie de kaposi avec une sérologie de l´herpès virus humain type 8 positive associée à un myélome multiple (MM) à IgG lambda stade I de pronostic défavorable. Nous rapportons à notre connaissance le 21^e^ cas kaposi-kahler. Ceci nous laisse penser à l´existence d´un variant kaposi herpès virus humain type 8 impliqué dans la physiopathologie du myélome multiple. D´où la nécessité d´élargir les études dans ce sens afin de trancher dans cette liaison exceptionnelle.

## Abstract (en)

Kaposi´s sarcoma is a tumor characterized by purple or brownish lesions affecting the skin. It is most commonly associated with human herpes virus type 8 (HHV-8) infection and may be secondary to malignant hemopathy, including lymphomas. We here report a new case of a very rare combination: Kaposi´s disease-multiple myeloma. The study involved Mr. aged 67 years, treated for Kaposi´s disease in the Department of Dermatology. Serological test for HHV-8 was positive; it was associated with stage I multiple myeloma IgG Lambda with a poor prognosis. We here report this 21^st^ case of Kaposi-Kahler in order to highlight Kaposi-human herpes 8 virus variant involved in the pathophysiology of multiple myeloma. More studies are needed in order to establish this exceptional link.

## Introduction

Le sarcome de kaposi (SK) ou maladie de kaposi est un processus prolifératif tumoral, caractérisé par des lésions violacées ou brunâtres sur la peau. Il peut également toucher les organes internes ou les muqueuses de la bouche, du nez et de l'anus. Il s´agit d´un cancer le plus souvent trouvé chez des personnes immunodéprimées (Sida) ou des patients sous traitements immunodépresseurs. Il est aussi lié à l´infection par l´herpès virus humain de type 8 (HHV-8) et peut être secondaire à une hémopathie maligne notamment les lymphomes [1]. Dans la littérature, seulement une vingtaine de cas d´association myélome multiple-kaposi ont été rapportés. Nous rapportons à travers notre observation un autre nouveau cas qui illustre cette association très rare.

## Patient et observation

**Figure 1:**
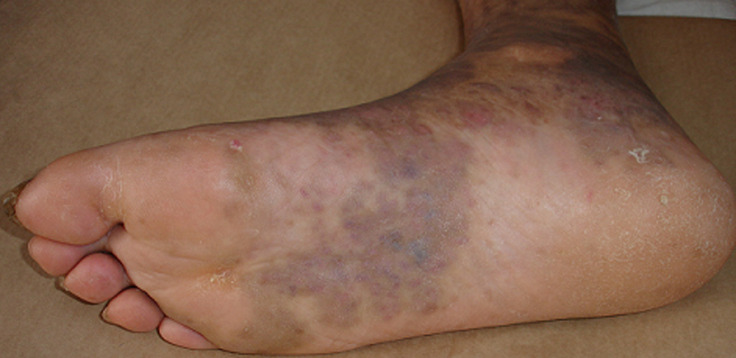
image clinique montrant des lésions violacées au niveau du pied

Mr MD âgé de 67 ans, suivi en dermatologie pour la maladie de kaposi. Le diagnostic de kaposi a été retenu devant le tableau clinique et biologique suivant: apparition des lésions papulonodulaires violacées au niveau des membres inférieurs (Figure 1); une biopsie cutanée qui a révélé la présence de cellules fusiforme; la sérologie HHV-8 qui a été positive et les sérologies VIH, hépatite B, C, cytomégalovirus ont été négatives. Le patient a bénéficié d´une radiothérapie et d´une chimiothérapie. Sept ans après, Mr MD a été admis au service de dermatologie de l´Hôpital Militaire d´Instruction Mohamed V de Rabat pour un œdème au niveau des lésions anciennes de kaposi. L´imagerie par résonance magnétique (IRM) du pied concerné a objectivé une anomalie de signale intéressant la graisse préachiléenne étendue au tendon d´achille sans lésion ostéo-articulaire, en rapport avec un processus inflammatoire. Lors d´un bilan général, l´hémogramme a montré une anémie normochrome normocytaire (Hb = 11,9g/dl), arégénérative. Le bilan biochimique a trouvé une créatinémie de 12mg/l, une urémie de 53mg/l, une calcémie de 104mg/l, une protéinurie de 24h de 0,09g/24. L´IRM médullaire n´a pas objectivé de lésions osseuses.

**Figure 2:**
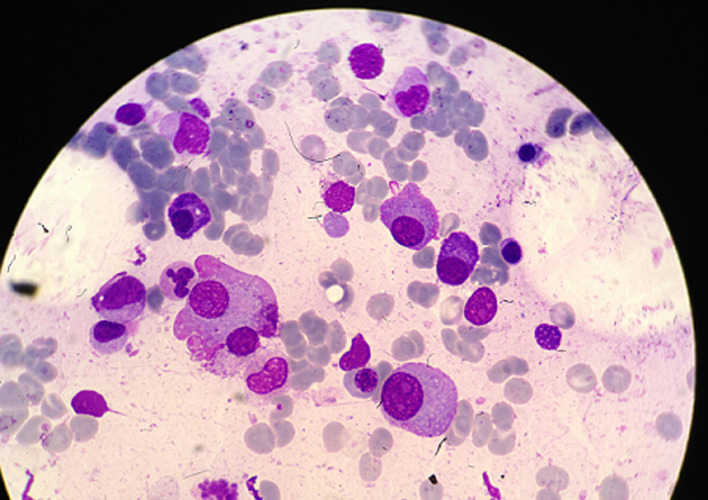
frottis médullaire coloré au MGG montrant des plasmocytes dystrophiques et immatures (GX100)

L´électrophorèse capillaire des protéines sériques a soulevé une découverte fortuite d´un pic étroit d´aspect monoclonal chiffré à 15,76g/l migrant dans la zone des gammaglobulines. L´immunosoustraction des protéines sériques a permis de typer l´immunoglobuline monoclonale (IgG lambda). L´immunofixation des protéines urinaires a montré la présence de chaînes légères libres monoclonales d´isotypie lambda en concentration inférieure à 10mg/l. Le bilan phosphocalcique a été normal (calcium corrigé: 93mg/l et phosphore: 32mg/L) ainsi que la fonction rénale (urée: 0,34g/l, créatinine à 12mg/l et la clairance selon la formule MDRD (modification of diet in renal disease) simplifiée à 61ml/min). La β_2_ microglobuline est élevée à 2,96mg/l. Un myélogramme a été réalisé qui a montré une moelle discrètement diluée avec un taux de plasmocytes à 07% fait d´éléments dystrophiques avec présence de formes immatures (Figure 2). Le dosage pondéral des Ig a été: (IgG=22,7g/l; IgA=0,83g/l; IgM=0,85g/l).

Le dosage des chaines légères libres kappa a été de 6,96mg/l et celui des chaines légères libres lambda de 43,83mg/l. Le rapport kappa libres et lambda libres de 0,16. L´étude cytogénétique moléculaire par FISH (nombre de cellules examinées: 400 plasmocytes purifiés CD138 +) a trouvé 80% des plasmocytes avec un remaniement IgH/FGFR3, équivalent moléculaire de la translocation t (4;14). De plus, 45% des plasmocytes présentent la trisomie du locus MM en 1q21 associée à la trisomie du locus SRD en 1p36 pour 14% des plasmocytes. En revanche, absence de délétion du locus P53 en 17p13. Ces résultats sont compatibles avec un myélome de pronostic défavorable. Il s´agit ainsi, d´un myélome multiple à IgG lambda stade I (selon la classification de Durie et Salmon).

## Discussion

L´association maladie de kaposi-hémopathie maligne est liée surtout aux lymphomes. La maladie de kaposi précède l´hémopathie dans seulement 15% des cas, elle apparaît simultanément dans 36% des cas et après dans 46% des cas [1]. Dans notre observation, il s´agit d´un patient traité pour SK et dont l´association concerne un myélome multiple de stade I et non un lymphome qui a été diagnostiqué secondairement. La maladie de kaposi est un type rare de cancer angiogénique multifocal permettant ainsi la transformation des cellules des vaisseaux en cellules cancéreuses qui se multiplient et se développent dans les tissus voisins. Elle est viro-induite par le HHV8, qui est observé dans presque tous les cas de SK. D´où son deuxième nom herpès-virus lié au sarcome de kaposi (HVSK). D´après la littérature, les séquences d´ADN apparentées au HHV8 sont détectées dans les lésions de kaposi, notamment dans les cellules fusiformes et les cellules endothéliales. La séroconversion se positive dans 33 mois en moyenne avant l´apparition des lésions de kaposi, permettant ainsi un diagnostic précoce en cas de suspicion [1,2]. L´infection par HHV-8 est rare en Amérique du Nord. Elle se manifeste plus souvent dans certains pays méditerranéens et est répandue en Afrique. Le HHV-8 se propage surtout par contact sexuel. Il peut aussi être transmis par le sang et la salive.

Bien que le HHV-8 reste dans le corps après l´infection, le système immunitaire le neutralise habituellement. Cependant, la plupart des personnes affectées par le HHV-8 n´éprouvent pas de symptômes. Ce virus ne semble pas causer de maladie chez la plupart des personnes en bonne santé. Les personnes qui sont atteintes d´un SK ont habituellement d´autres problèmes de santé qui affaiblissent leur système immunitaire, tels que: le syndrome d´immunodéficience acquise (Sida), la prise de médicaments immunosuppresseurs (après une greffe d´organe par exemple), une maladie du sang comme la maladie de Castleman [3-5]. Notre malade a développé un SK sans être immunodéprimé, avec une sérologie VIH négative et sans antécédent de prise médicamenteuse. Le HHV-8 touche majoritairement les lymphocytes B et les cellules fusiformes endothéliales, mais également d´autres types cellulaires. Ainsi il code pour des analogues de cytokines humaines, notamment l´IL-6. En effet l´interleukine-6 est une cytokine à action paracrine connue pour son rôle majeur dans l´émergence du clone tumoral plamocytaire. Cette cytokine, via l´interaction avec son récepteur spécifique, l´IL-6Rα, entraîne l´homodimérisation de la chaîne transductrice Gp130 qui déclenche une cascade de signaux associant la voie des Jak/STAT à la voie Ras/MAP kinase.

L´IL-6 délivre ainsi un signal de survie et de prolifération aux plasmocytes tumoraux; hors que les homologues viraux d´IL-6 sont capables d´activer directement la chaîne gp130, indépendamment de l´IL-6Rα [2,6,7]. Ces observations laissent croire que l´inflammation contribue dans le développement des lésions cutanées angiomatoses du SK ainsi que la stimulation excessive du clone B plasmocytaire. Toutefois, le KSHV, qui est retrouvé dans tous les cas de sarcome de kaposi, est également associé à deux syndromes lymphoprolifératifs B rares où l´IL-6 joue un rôle prépondérant: le lymphome des cavités et la maladie de Castleman. Dans ce dernier cas, il existe même une corrélation entre la détection d´IL-6 virale et la gravité de la maladie [8,9]. En 1997, l´équipe de J. Berenson (Los Angeles, CA, USA) a suggéré que KSHV pourrait être un facteur causal du myélome multiple. En effet, elle a pu mettre en évidence la présence du virus dans des cultures de plusieurs semaines de cellules dendritiques stromales médullaires (CD68+, CD83+, Fascine+) chez tous les patients atteints de myélome multiple et chez une partie des patients atteints de MGUS (monoclonal gammopathy of undetermined significance) alors qu´il était indécelable dans les aspirations médullaires au moment de la mise en culture.

La même équipe a mis en évidence la présence de KSHV dans la moelle osseuse de certains patients atteints de MGUS (3 sur 5 testés) qui pourrait être impliqué dans la transformation de ces MGUS en MM. La culture cellulaire du virus HHV-8 ainsi que la détection de l´ADN viral au niveau des cellules myélomateuse n´ont pas été faites dans notre cas vu qu´elles ne peuvent être pratiquées que dans des laboratoires spécialisés [9]. Toutes ces données restent très controversées. Le SK est un réel cancer angiogénique provoqué par l´HHV-8, ou un processus réactionnel, médié par les facteurs de croissance et les cytokines notamment l´IL-6. Notre malade avait un MM de stade I avec des anomalies génétiques de mauvais pronostic associée aux lésions angiomatoses cutanées au niveau du pied, cette liaison nous laisse penser à l´existence d´un variant KHHV-8 impliqué dans la physiopathologie du MM. D´où la nécessité d´élargir les études dans ce sens afin de trancher dans cette liaison exceptionnelle kaposi-kahler.

## Conclusion

Nous rapportons à notre connaissance le 21^e^ cas, associant le sarcome de kaposi-MM, avec une sérologie HHV8 positive. Il s´agit donc d´une association exceptionnelle qui pourrait prouver le rôle majeur de l´HHV-8 dans: la survenue; l´évolution clinique et l´apparition de nouvelles molécules traitantes du MM.
